# The mediating effect of social relationships on the association between socioeconomic status and subjective health – results from the Heinz Nixdorf Recall cohort study

**DOI:** 10.1186/1471-2458-12-285

**Published:** 2012-04-17

**Authors:** Nico Vonneilich, Karl-Heinz Jöckel, Raimund Erbel, Jens Klein, Nico Dragano, Johannes Siegrist, Olaf von dem Knesebeck

**Affiliations:** 1 Department of Medical Sociology and Health Economics, University Medical Center Hamburg-Eppendorf, Hamburg, Germany; 2 Institute of Medical Informatics, Biometry and Epidemiology, University Duisburg-Essen, Essen, Germany; 3 Clinic for Cardiology, University Clinic Essen, Essen, Germany; 4 Department of Medical Sociology, Heinrich Heine University Düsseldorf, Düsseldorf, Germany

## Abstract

**Background:**

Socioeconomic status (SES) is an important determinant of population health. Explanatory approaches on how SES determines health have so far included numerous factors, amongst them psychosocial factors such as social relationships. However, it is unclear whether social relationships can help explain socioeconomic differences in general subjective health. Do different aspects of social relationships contribute differently to the explanation? Based on a cohort study of middle and older aged residents (45 to 75 years) from the Ruhr Area in Germany our study tries to clarify the matter.

**Methods:**

For the analyses data from the population-based prospective Heinz Nixdorf Recall (HNR) Study is used. As indicators of SES education, equivalent household income and occupational status were employed. Social relations were assessed by including structural as well as functional aspects. Structural aspects were estimated by the Social Integration Index (SII) and functional aspects were measured by availability of emotional and instrumental support. Data on general subjective health status was available for both baseline examination (2000–2003) and a 5-year follow-up (2006–2008). The sample consists of 4,146 men and women. Four logistic regression models were calculated: in the first model we controlled for age and subjective health at baseline, while in models 2 and 3, either functional or structural aspects of social relationships were introduced separately. Model 4 then included all variables. As former studies indicated different health effects of SES and social relations in men and women, analyses were conducted with the overall sample as well as for each gender alone.

**Results:**

Prospective associations of SES and subjective health were reduced after introducing social relationships into the regression models. Percentage reductions between 2% and 30% were observed in the overall sample when all aspects of social relations were included. The percentage reductions were strongest in the lowest SES group. Gender specific analyses revealed mediating effects of social relationships in women and men. The magnitude of mediating effects varied depending on the indicators of SES and social relations.

**Conclusions:**

Social relationships substantially contribute to the explanation of SES differences in subjective health. Interventions for improving social relations which especially focus on socially deprived groups are likely to help reducing socioeconomic disparities in health.

## Background

Numerous studies show that socioeconomic status (SES) affects people’s health status. A social gradient can be identified for different health indicators, for example mortality, morbidity or self-rated health: each step down on the social ladder is associated with increasing health risks [1-3].

As with health, SES has also been found to affect social relations. Different SES indicators have been associated with social relationships in previous studies: poor social relations are more likely to occur in low SES groups [4,5]. A recent study using the same data as our present analysis showed that poorer social relations were more frequent among lower status groups [6]. For example this study revealed that persons with low education had an odds ratio (OR) of 2.1 to report social isolation compared to the group with highest education. When SES was measured by income, low income groups had an OR of 2.4 to report social isolation.

When studying social relations, generally qualitative and quantitative aspects are differentiated. Quantitative or structural aspects of social relations such as number, frequency and intensity of social contacts are widely used in social-epidemiologic research and can be measured by a well-established index, the Social Integration Index (SII) [7]. It includes information on marital status, number of close contacts and participation in volunteer organisations. Functional or qualitative aspects of social relations are assessed by means of social support. Here, a distinction is especially made between emotional and instrumental support [8]. Emotional support includes help in decision-making, understanding and someone to discuss daily problems with, while instrumental support focuses on practical help or financial aid.

Both aspects of social relations have repeatedly been associated with different health indicators, including self-rated health [8-16]. Also, social support has been found to be associated with mental health [4,17-19]. A lack of social ties has repeatedly been found to increase mortality from different causes of death [20]. A recently published review concludes that the influence of social relationships on mortality risks is highly comparable with biomedical and behavioural risk factors [21].

Explanatory approaches on health inequalities have so far included behavioural, material and psychosocial factors [3,22,23]. Among these psychosocial factors are social relations. Only few studies systematically examined the explanatory effect of social relationships on the association between SES and health. In an early work on different contributing factors for explaining socioeconomic differences in health, Marmot and colleagues found a slight explanatory contribution of social relations for the social gradient in self-reported health, waist-hip ratio and psychological well-being [3]. A US study using cross-sectional data from the National Health Interview Survey indicated only little evidence for a contribution of social support and social integration [24]. Another US study showed that psychosocial factors were independent determinants of health but no explanatory factors for SES effects on subjective health [25]. A study among older Danes found no mediating effect of social relations on socioeconomic differences in the onset of disability [26]. A German study revealed weak mediating effects among the aged [12]. Emotional support contributed only little to explaining educational differences in general subjective health in a European comparison [27]. Overall, results on mediating effects have been ambiguous as no clear results regarding the possible mediating effect of social relations on the association of SES and health were found in former studies.

The following analyses are drawn upon a population-based cohort study, the Heinz-Nixdorf Recall (HNR) study, which was carried out in the Ruhr Area. The Ruhr Area has several specific characteristics. It is the largest metropolitan area in Germany with several cities, of which three are included in the study. It is an area in transition, which used to be dominated by an industrial productive sector, specialised on coal and steel. With the closing down of most of this industrial sector, a shift to the third sector took place. This process came along with rising unemployment. At the same time, a process of ‘suburbanisation’ set in. High and middle income families moved into the more rural surrounding areas, leaving an older, poorer and mostly childless population behind. The Ruhr Area as the second biggest metropolitan area in Europe will lose about 7% of its population (about 374,000 inhabitants) due to this process of suburbanisation between 1998 and 2015 [28]. Therefore, we suspect that in areas undergoing such structural changes and in times of greater uncertainty, social relationships become even more important as a source of support and might play an even greater role in explaining negative health effects of low SES.

Against this background we examine whether social relationships can help explain socioeconomic differences in subjective health in a region characterised by structural changes, as stated in the hypothesis of differential exposure [29]. Because it can be assumed that the inconsistency of the results in former studies may be due to the use of different indicators, SES is measured by three indicators (education, income and occupational status). Also qualitative as well as quantitative aspects of social relations are covered. Moreover, as earlier studies showed that SES and social relationships have different health effects in men and women [4,30-32], our analyses are based on the overall sample as well as on the subsample of men and women separately.

## Methods

### Study population

Data stem from the Heinz Nixdorf Recall (HNR) Study. This prospective population-based cohort study was conducted in an industrialised urban region (Ruhr Area) in Western Germany. Rationale, design and methods of this study have been described in detail elsewhere [33,34]. The original sample contains 4,814 middle and older aged residents of the Ruhr Area between 45 and 75 years, as one of the main study goals was to improve prediction of coronary heart disease by combining already established with new cardiovascular risk factors. This corresponds to a response rate of 55.8% at baseline. Respondents were recruited from three adjacent cities (Bochum, Essen and Muelheim/Ruhr). Baseline examinations started in 2000 and ended in 2003, the 5 year follow-up took place from 2005 through 2008. All indicators used in the following analyses were assessed at baseline as part of a social risk factor assessment by face-to-face interviews and paper-and-pencil questionnaires. Data on general subjective health is also available from a 5-year follow-up. Only those respondents of whom information on subjective health was available at both baseline examination and follow-up were included in the analyses. This restriction resulted in an effective sample size of N = 4,146 individuals (86.1% of the baseline sample).

### Measures

#### *Socioeconomic status (SES)*

We used three SES measures, as income, education and occupational status can all have different meanings and thus also have different impact on population health [3,35]. Education was classified according to the International Standard Classification of Education (ISCED) as total years of formal education, combining school and vocational training [36]. 18 and more years of formal education are equivalent to a university degree, while 10 or fewer years correspond to a basic school degree and no vocational training. Income was measured by equivalent household income including information on disposable income and size of household with number of adults and children according to OECD criteria, the so called ‘OECD-modified scale’ [37]. The respondent was attributed with a weight of 1, while every other household member was given a weight of 0.5. Data on occupational status of respondents includes last job before retirement. As the study population consists of mainly older age-groups, including a higher percentage of retired persons, information on job status combines actual job and, in case of retirement, last job before retirement. A high percentage of women in the study population never had regular employment, which is common in this specific age-group in Germany. Therefore and because of missing information no occupational status could be computed for 669 (32%) women (compared to missing information of 82 (4%) men). Occupational status was measured by the International Standard Classification of Occupation (ISCO-88) scale. The original data was manually transformed into a hierarchical general four-level job scale, according to the original ISCO-88 job classification, with the first level indicating ‘Manager and professionals’, the second ‘Technicians and associate professionals’, the third ‘Qualified employees’ and the fourth covering ‘Unskilled employees’ [38,39].

#### *Social integration index*

The SII, which was originally developed by Lisa F. Berkman [40], captures quantitative aspects of social relations and thereby makes the degree of an individual’s social integration measurable. This multidimensional measure includes the marital status respectively living with a partner, the number of contacts with close ties (including family members and friends) as well as the affiliation with voluntary associations. Each of these three domains score from 0 to 2 depending on the grade of integration: marital status or cohabitation was scored 2, all else 0; number of close ties was scored 0 for 0–2 contacts, 1 for 3–11 contacts and 2 for 12 or more contacts; participation in voluntary associations was scored 0 for no participation, 1 for participation in one association, and 2 for participation in more than one voluntary association. The total score ranges from 0 to 6. In order to obtain the original SII, this score was categorised into four levels of integration: level I (Score 0–1), II (Score 2 and 3), III (Score 4 and 5) and IV (Score 6) [40].

#### *Social support*

Measures of social support include perceived instrumental and emotional support. Both were assessed by a German adaptation of the New Haven Established Populations for Epidemiologic Studies of the Elderly (EPESE) questionnaire [9]. Instrumental support refers to help available in daily tasks, for example shopping, cooking or washing. Emotional support includes having someone to talk to, someone to discuss problems with or someone who helps making difficult decisions. First, questions of both support measures assessed the perceived availability of someone to help and the presence of one or more persons to approach when problems were experienced. In a second step, the respondents were asked who actually needed support in the last 12 months and whether this support was available and appropriate. Based on the combination of this information, four categories were built: ‘support available but not needed’, ‘support appropriate’, ‘support inappropriate’ and ‘support needed but not available’ [6].

#### *Subjective health*

The general subjective health status, assessed at both baseline and 5 year follow-up examination, was used as health indicator. Subjective health is a widely accepted measure for health, which has been linked to mortality and morbidity in a wide range of population studies [41]. In the HNR study it was assessed by one question (‘How would you, referring to the last twelve months, describe your overall health status?’) on a 5-point Likert-scale (‘very good’, ‘good’, ‘moderate’, ‘poor’ and ‘very poor’) at baseline and at follow-up examination. For the purpose of logistic regression analyses, the follow-up health measure was used as outcome and has therefore been dichotomised, with ‘poor’ or ‘very poor’ representing poor general subjective health status.

### Statistical analysis

All analyses are based on prospective multiple logistic regressions. Results are displayed by OR and 95% confidence intervals.

As mentioned earlier, we focus on the mediating effect of social relations on the association of SES and subjective health. A mediator is a variable, which can partly explain the association between a focal independent and a dependent variable. Analytic and theoretical considerations regarding mediating effects have been stated by Baron and Kenny [42]. In a regression model, the association between predictor and criterion should be reduced or even diminished after the mediator is introduced into the model. The role of the mediator is detected by calculating a set of multiple logistic regression models. In a first basic model ORs are calculated for the association between each of the SES indicators and subjective health at follow-up, controlling for age and initial subjective health. In a second model, SII is introduced as a possible mediating variable. The third model includes emotional and instrumental support as measures of qualitative aspects of social relations but excludes SII. A fourth model then comprises all before mentioned variables. These four models are calculated for each SES indicator with an overall sample as well as for both genders separately. Mediating effects are represented by percentage reductions. Percentage reductions in point estimates are calculated by using the following formula: $\left(\left[{\text{OR}}_{\text{Model }1}-{\text{OR}}_{\text{Model }1+\text{social relations}}\right]/\left[{\text{OR}}_{\text{Model }1}-1\right]\right)\times 100$[43]. Percentage change is displayed when the precondition of a statistically significant OR in the first model is satisfied (p < 0.05). All analyses are conducted with the statistical programme package PASW 18.0.

## Results

Descriptive characteristics of the study population are shown in Table 1. As mentioned above, only those cases with information on general subjective health at baseline and follow-up were included in the analyses (N = 4,146). Chi-square statistics were included in order to reveal significant differences between the genders. P-values of the Chi-square tests are displayed. Slight changes between baseline examination and follow-up occurred in self-rated health: while at baseline about 15% rated their health as poor or very poor, 5 years later this number rose to 17%. Changes in subjective health were stronger in men than in women.

**Table 1:** Sample characteristics (Heinz Nixdorf Recall Study)

		**Overall** **N (%)**	**Men** **N (%)**	**Women** **N (%)**	**p (Chi²)**
**Total sample**		**4,146**	**2,049 (49.5)**	**2,097 (50.5)**	
***Variables****(no. of missings)*					
**Age** (0)	Mean [SD]	58.8 [7.7]	58.8 [7.6]	58.7 [7.7]	
**Years of Education** (5)	<=10 years	418 (10.1)	85 (4.2)	333 (15.9)	0.000
	11-13	2,314 (55.8)	964 (47.1)	1,350 (64.4)	
	14-17	940 (22.7)	701 (34.2)	239 (11.4)	
	= > 18 years	472 (11.4)	298 (14.6)	174 (8.3)	
**Household equivalent income per month** (260)	<1,000€ (very low)	896 (23.0)	372 (18.9)	524 (27.3)	0.000
	1,000-1,500€ (low)	1,285 (33.0)	622 (31.6)	663 (34.5)	
	1,500-2,000€ (average)	923 (23.7)	515 (26.1)	408 (21.3)	
	>2,000€ (high)	785 (20.2)	461 (23.4)	324 (16.9)	
**Occupational Status** (754)	Unskilled employees/ workers	580 (17.1)	196 (10.0)	384 (26.9)	0.000
	Qualified employees/ workers	1,401 (41.3)	800 (40.7)	601 (42.1)	
	Technicians and associate professionals	796 (23.4)	518 (26.3)	278 (19.5)	
	Manager and Professionals	618 (18.2)	453 (23.0)	165 (11.6)	
**Social Integration Index** (68)	Level I (isolation)	273 (6.7)	73 (3.6)	200 (9.7)	0.000
	Level II	1,656 (40.6)	757 (37.6)	899 (43.5)	
	Level III	1,943 (47.6)	1.076 (53.4)	867 (42.0)	
	Level IV	209 (5.1)	109 (5.4)	100 (4.8)	
**Instrumental support** (30)	Support available but not needed	1,278 (30.8)	669 (32.8)	609 (29.3)	0.000
	Support appropriate	2,357 (57.2)	1,165 (57.2)	1,192 (57.3)	
	Support inappropriate	171 (4.1)	55 (2.7)	116 (5.6)	
	Support needed but not available	313 (7.5)	148 (7.3)	165 (7.9)	
**Emotional support** (24)	Support available but not needed	458 (11.0)	289 (14.2)	169 (8.1)	0.000
	Support appropriate	3019 (72.8)	1,443 (70.7)	1,576 (75.6)	
	Support inappropriate	311 (7.5)	101 (5.0)	210 (10.1)	
	Support needed but not available	337 (8.1)	207 (5.0)	130 (6.2)	
**Subjective Health**	***Baseline****(3)****:*** Very good/ good/ moderate	3,509 (84.6)	1,810 (88.3)	1,699 (81.0)	0.000
	Poor/ very poor	637 (15.4)	239 (11.7)	398 (19.0)	
	***Follow-up****(0)****:*** Very good/ good/ moderate	3,426 (82.6)	1,742 (84.9)	1,684 (80.3)	0.000
	Poor/ very poor	723 (17.4)	310 (15.1)	413 (19.7)	

Significant gender differences were found in nearly all variables except for age and emotional support. Women reported a lower SES: they had significantly fewer years of education, reported lower household equivalent income per month and also a lower occupational status than men. Furthermore, women reported themselves to be less socially integrated as indicated by the SII score. Also, women were more likely to rate their health as poor or very poor at both baseline examination and follow-up. These differences underline the above mentioned argument for gender-specific analyses.

Multivariate analyses revealed a social gradient for subjective health in a longitudinal perspective: the lower a person’s SES at baseline (measured by education, income, and occupational status), the higher were the odds for reporting poor or very poor subjective health at 5 year follow-up, after controlling for subjective health at baseline in the overall sample (Table 2). Generally, the lowest socioeconomic groups showed highest risks for poor subjective health.

**Table 2:** **Socioeconomic status at baseline and subjective health at follow-up: Odds ratios**^**1**^**, 95% confidence intervals (CI) and percentage change**^**2**^**(N = 4,146)**

***Overall sample***	**Subjective health (poor/rather poor)**											
	**Education**(Reference Category: > 18 years)			**Change** (%)^2^	**Equivalent household income**(Reference Category: high)			**Change** (%)^2^	**Occupational status**(Reference category: Manager and Professionals)			**Change** (%)^2^
**Model 1:**	14-17 years	1.30	(0.90-1.89)		average	1.04	(0.77-1.39)		Technicians	**1.49**	(1.06-2.09)	
adjusted for age, gender and general health status at baseline	11-13 years	**1.50**	(1.07-2.10)		low	1.10	(0.84-1.45)		Qualified employees	**1.53**	(1.12-2.08)	
	≤10 years	**1.79**	(1.19-2.69)		very low	**1.64**	(1.23-2.17)		Unskilled	**1.90**	(1.34-2.69)	
**Model 2:**	14-17 years	1.27	(0.88-1.85)		average	1.04	(0.76-1.37)		Technicians	**1.48**	(1.06-2.10)	−2.0
Model 1 additionally adjusted for Social Integration Index	11-13 years	**1.43**	(1.02-2.01)	−14.0	low	1.10	(0.82-1.42)		Qualified employees	**1.48**	(1.09-2.03)	−9.4
	≤10 years	**1.67**	(1.10-2.51)	−15.2	very low	**1.54**	(1.16-2.05)	−15.6	Unskilled	**1.77**	(1.24-2.52)	−14.4
**Model 3:**	14-17 years	1.31	(0.90-1.90)		average	1.01	(0.75-1.36)		Technicians	**1.46**	(1.04-2.05)	−6.1
Model 1 additionally adjusted for instrumental and emotional support	11-13 years	**1.48**	(1.06-2.09)	−4.0	low	1.06	(0.81-1.40)		Qualified employees	**1.46**	(1.07-1.99)	−13.2
	≤10 years	**1.73**	(1.15-2.62)	−7.5	very low	**1.55**	(1.17-2.06)	−14.1	Unskilled	**1.82**	(1.28-2.59)	−8.9
**Model 4:**	14-17 years	1.28	(0.88-1.87)		average	1.01	(0.75-1.36)		Technicians	**1.46**	(1.04-2.06)	−6.1
Model 1 additionally adjusted for all three indicators of social relations	11-13 years	**1.42**	(1.01-2.01)	−16.0	low	1.06	(0.80-1.41)		Qualified employees	**1.43**	(1.04-1.95)	−18.9
	≤10 years	**1.64**	(1.08-2.49)	−19.0	very low	**1.47**	(1.10-1.96)	−26.6	Unskilled	**1.71**	(1.20-2.44)	−21.1

^1^Significant odds ratios are bold (p < 0.05).

^2^Percentage change in OR (Model 1 compared separately at a time with Model 2, Model 3 and Model 4) using $\left(\left[{\text{OR}}_{\text{Model }1}-{\text{OR}}_{\text{Model }1+\text{social relations}}\right]/\left[{\text{OR}}_{\text{Model }1}-1\right]\right)\times 100$. Percentage change is displayed when OR is statistically significant in Model 1 (p < 0.05).

The mediator analyses indicated a mediating effect of social relations on the association between SES and self-rated health. Percentage reductions were found for all SES indicators after the introduction of social relations, even though these reductions varied in strength. When education was used as SES measure, percentage reductions ranged between 2% and about 18%. Similar results were found for occupational status. The introduction of social relations into the association of equivalent household income and subjective health led to strongest reductions: here social relations explained between 15% and 30% of the association.

Generally, strongest percentage reductions were found in Model 4, when structural and functional aspects of social relations were introduced into the analyses simultaneously. Percentage reductions ranged between 6% and 30%. Percentage reductions in Model 2 varied between 2% and about 16%, while in Model 3 they varied between 2% and 17% in the overall sample.

In men, only the lowest income and occupational categories are significantly associated with poor subjective health in model 1 (Table 3). Therefore, percentage reductions were only calculated for these two SES groups. After the introduction of social relations into the multiple logistic regression models, percentage reductions of up to 15% were observed.

**Table 3:** **Socioeconomic status at baseline and subjective health at follow-up, men: Odds ratios**^**1**^**, 95% confidence intervals (CI) and percentage change**^**2**^**(N = 2,049)**

***Men***	**Subjective health (poor/rather poor)**											
	**Education**(Reference Category: > 18 years)			**Change** (%)^2^	**Equivalent household income**(Reference Category: high)			**Change** (%)^2^	**Occupational status**(Reference category: Manager and Professionals)			**Change** (%)^2^
**Model 1:**	14-17 years	1.16	(0.73-1.85)		average	0.98	(0.66-1.47)		Technicians	1.45	(0.97-2.19)	
adjusted for age and general health status at baseline	11-13 years	1.43	(0.92-2.22)		low	0.97	(0.66-1.42)		Qualified employees	1.33	(0.91-1.95)	
	≤10 years	1.29	(0.63-2.66)		very low	**1.84**	(1.24-2.73)		Unskilled	**1.97**	(1.22-3.19)	
**Model 2:**	14-17 years	1.12	(0.70-1.78)		average	1.01	(0.68-1.52)		Technicians	1.44	(0.95-2.17)	
Model 1 additionally adjusted for Social Integration Index	11-13 years	1.36	(0.87-2.11)		low	0.98	(0.66-1.44)		Qualified employees	1.26	(0.86-1.84)	
	≤10 years	1.19	(0.58-2.46)		very low	**1.83**	(1.23-2.73)	−2.4	Unskilled	**1.85**	(1.13-3.01)	−9.3
**Model 3:**	14-17 years	1.14	(0.58-2.52)		average	0.97	(0.65-1.45)		Technicians	1.41	(0.94-2.13)	
Model 1 additionally adjusted for instrumental and emotional support	11-13 years	1.38	(0.89-2.15)		low	0.95	(0.64-1.40)		Qualified employees	1.26	(0.86-1.85)	
	≤10 years	1.21	(0.58-2.52)		very low	**1.76**	(1.18-2.62)	−9.5	Unskilled	**1.92**	(1.18-3.13)	−5.2
**Model 4:**	14-17 years	1.10	(0.69-1.76)		average	1.00	(0.67-1.50)		Technicians	1.41	(0.93-2.13)	
Model 1 additionally adjusted for all three indicators of social relations	11-13 years	1.32	(0.85-2.06)		low	0.96	(0.65-1.42)		Qualified employees	1.20	(0.82-1.77)	
	≤10 years	1.13	(0.54-2.35)		very low	**1.74**	(1.16-2.60)	−11.9	Unskilled	**1.80**	(1.10-2.96)	−17.5

^1^Significant odds ratios are bold (p < 0.05).

^2^Percentage change in OR (Model 1 compared separately at a time with Model 2, Model 3 and Model 4) using ([OR_Model 1_**-**OR_Model 1+social relations_]/[OR_Model 1_-1]) x 100. Percentage change is displayed when OR is statistically significant in Model 1 (p < 0.05).

In women, significantly higher risks for poor subjective health at follow-up were found for lowest SES groups (Table 4). Contribution of social relations to explain these health inequalities considerably differed depending on the indicators used. Percentage reductions varied between 2% and 50%.

**Table 4:** **Socioeconomic status at baseline and subjective health at follow-up, women: Odds ratios**^**1**^**, 95% confidence intervals (CI) and percentage change**^**2**^**(N = 2,097)**

***Women***	**Subjective health (poor/rather poor)**											
	**Education**(Reference Category: > 18 years)			**Change** (%)^2^	**Equivalent household income**(Reference Category: high)			**Change** (%)^2^	**Occupational status**(Reference category: Manager and Professionals)			**Change** (%)^2^
**Model 1:**	14-17 years	1.65	(0.88-3.07)		average	1.11	(0.72-1.73)		Technicians	1.64	(0.89-3.00)	
adjusted for age and general health status at baseline	11-13 years	1.64	(0.96-2.79)		low	1.22	(0.82-1.82)		Qualified employees	**1.89**	(1.09-3.29)	
	≤10 years	**2.00**	(1.11-3.59)		very low	**1.50**	(1.00-2.26)		Unskilled	**2.00**	(1.13-3.54)	
**Model 2:**	14-17 years	1.65	(0.88-3.09)		average	1.07	(0.69-1.67)		Technicians	1.67	(0.91-3.09)	
Model 1 additionally adjusted for Social Integration Index	11-13 years	1.59	(0.93-2.72)		low	1.19	(0.79-1.78)		Qualified employees	**1.95**	(1.12-3.40)	+6.7
	≤10 years	**1.92**	(1.07-3.48)	−8.0	very low	1.36	(0.90-2.05)	−28.0	Unskilled	**1.92**	(1.08-3.43)	−8.0
**Model 3:**	14-17 years	1.76	(0.93-3.33)		average	1.06	(0.68-1.65)		Technicians	1.59	(0.87-2.93)	
Model 1 additionally adjusted for instrumental and emotional support	11-13 years	1.68	(0.97-2.91)		low	1.15	(0.77-1.73)		Qualified employees	**1.81**	(1.04-3.14)	−9.0
	≤10 years	**2.01**	(1.10-3.67)	+1.0	very low	1.40	(0.93-2.12)	−20.0	Unskilled	**1.89**	(1.07-3.36)	−11.0
**Model 4:**	14-17 years	1.75	(0.92-3.32)		average	1.03	(0.66-1.60)		Technicians	1.63	(0.88-3.01)	
Model 1 additionally adjusted for all three indicators of social relations	11-13 years	1.64	(0.95-2.85)		low	1.13	(0.76-1.70)		Qualified employees	**1.86**	(1.07-3.25)	−3.4
	≤10 years	**1.97**	(1.08-3.62)	−3.0	very low	1.28	(0.84-1.94)	−44.0	Unskilled	**1.84**	(1.03-3.28)	−16.0

^1^Significant odds ratios are bold (p < 0.05).

^2^Percentage change in OR (Model 1 compared separately at a time with Model 2, Model 3 and Model 4) using ([OR_Model 1_**-**OR_Model 1+social relations_]/[OR_Model 1_-1]) x 100. Percentage change is displayed when OR is statistically significant in Model 1 (p < 0.05).

## Discussion

This is one of the first studies that systematically examined the mediating effect of social relationships on the association between SES and subjective health using data from a 5 year follow-up. Our results indicate a mediating effect of social relationships, i.e. social relations contribute to the explanation of socioeconomic inequalities in subjective health. When measures of social relations were introduced as mediators into the regression models, percentage reductions of the odds ratios between 2% and 30% were observed in the overall sample. In most cases percent reductions exceeded 10%. If the associations between indicators of SES and general subjective health were to be independent of social relations, a variation in effect size would not have been found after the introduction of SII, emotional and instrumental support into the regression models.

Former studies have not consistently revealed such a mediating effect of social relationships on health. Some studies found only slight mediating effects of social relations [3,12], while others showed no contribution of social relations to the explanation of health inequalities [24,25]. However, these studies differ in terms of study design, measurement of social relations and health as well as study region.

Regarding gender differences, SES indicators are differently associated with subjective health in men and women. On the one hand, a low equivalent household income leads to stronger OR for poor subjective health in men than in women. On the other hand, less than 10 years of education are more strongly related to poor subjective health in women than in men (see Tables 3 and 4). Especially with regard to the specific age-group of the study, which is characterised by a lower degree of labour participation in women, one could imagine that income is of higher importance to men, as it might more directly reflect their success as “breadwinners”. The social status of women in this age-group might be more accurately assessed by their educational background. This might help to explain, why these indicators are differently associated with health in men and women. The mediator analyses revealed similar results as mediating effects of social relationships were detected for both men and women, though varying in effect size. For example, when equivalent household income was used as SES measure, a percentage reduction of up to 50% was found in women, while in men the percentage reduction was 15% in the lowest income group (see Tables 3 and 4). Due to a reduced sample size in the gender-specific analyses, the effects in the first basic model rarely reached significance. Therefore, percentage reductions were not calculated in most cases. Generally, the introduction of social relations reduces the association of SES and subjective health in women and in men. Two exceptions can be found in women (see Table 4). The introduction of instrumental and emotional support (Model 3) leads to a small percentage increase of the respective OR in women with less than 10 years of education. Secondly, the introduction of the SII leads to a 7% increase of the OR for poor health among female qualified employees compared to managers and professionals. As these increases are minor and no particular pattern can be observed, we are careful in drawing conclusions from these findings. In our view it is important to further investigate gender differences in the association between SES, social relations and health in future studies, because we are far from understanding the particular mechanisms in men and women [19].

Regarding the explanatory contribution of social support and social integration, results are inconsistent. Overall, the simultaneous introduction of both aspects of social relations leads to largest percentage reductions. However, it remains unclear, which aspect of social relations contributes most to the explanation of inequalities in subjective health. For example when SES is measured by education, the introduction of the SII alone leads to percentage reductions of about 15%, while the introduction of instrumental and emotional support shows marginal percentage reductions of between 2% and 6% (see Table 2). When SES is measured by occupational status, the introduction of social support leads to stronger percentage reductions in technicians and qualified employees than does the introduction of SII, while for the unskilled the opposite is true. We additionally analysed in which way the two indicators of functional aspects of social relations, emotional and instrumental support, contribute differently to the explanation of socioeconomic inequalities in subjective health. Hence, emotional and instrumental support were introduced separately into logistic regression models (results not shown). The explanatory contribution proved to be very similar, with no clear pattern of differences regarding the explanatory contribution of instrumental and emotional support.

Earlier research has led to a vast body of evidence showing different associations of functional and structural aspects of social relations with different health indicators [4,9,11,21,44]. While structural aspects of social relations may facilitate the availability of help, social support might more directly affect health behaviour and psychological mechanisms such as feelings of self-esteem and coping [9,15,45]. It has been highlighted that especially for ones feeling of accessibility of support and its effect on health it is important to distinguish between perceived and actually received support [44]. Stansfeld and Fuhrer have developed several models to show how different facets of social relations may influence population health [11]. In a meta-analytic review Holt-Lunstad and colleagues showed that especially a multidimensional assessment of social-relations led to strongest associations with mortality-risks, as they included the different pathways by which social relations influence health and mortality [21].

Regarding the three SES indicators, similar results in the strength of the associations can be observed. Generally, the lowest SES groups have the highest risks of reporting poor subjective health. Moreover, in this group percentage reductions are largest when social relations are introduced into the logistic regression models. This is true for income, education as well as for occupational status.

In interpreting the presented results, several methodological aspects should be considered. It is a strength of our analyses that they are based on a cohort study. So far no study has been able to draw conclusions on the mediating effect of social relations on the association of SES and subjective health in a longitudinal perspective. Furthermore, special emphasis was put on quality control of data collection and data handling in the HNR study, as evidenced by external certification [33]. Complex measures of social support indicators were used. When constructing the items for measuring support, both availability as well as adequacy of support were considered, as proposed in earlier research [18].

On the other hand our results are limited as they are all based on self-reported data and do not include objectively measured health indicators. Therefore, a possible bias can not be ruled out. Also the longitudinal design is limited as we refer to one 5-year follow-up and include only two measurement points. As expected, there is only little variation in subjective health in a 5-year period. Moreover, we did not calculate significances of mediator effects by using the Sobel-Test as multiple mediators were included in our model. Another restriction is the high number of missing information on occupational status, especially in women (N = 669). For those cases, no information on actual job status or occupational status before retirement was available. This might lead to bias in the results for this SES category. Furthermore, analyses draw on a specific study population, namely a sample of residents of the Ruhr Area aged 45 to 75 years. As noted earlier, the Ruhr Area is a region in transition. It is therefore possible, that in such uncertain times of change, social relationships play a particularly important role in protecting individuals from negative health effects of socioeconomic hardship. This may be one reason why mediating effects of social relationships found in our study are more consistent than in former studies. Therefore, our results cannot be generalised to the overall German population but maybe to populations living under similar conditions.

## Conclusions

Research in social epidemiology often focuses either on health effects of social relationships or on socioeconomic differences in health. Our findings indicate that these two important social determinants of population health are linked. Social relations seem to play a role in explaining increased health risks of low SES groups. Accordingly, an improvement of social relations can help lower SES groups to enhance their subjective well-being and health. It has been noted in earlier studies, that interventions for improving social relations might be more efficient when they concentrate on deprived populations [43]. Social relations can positively affect health by promoting a healthier lifestyle, by helping to cope with psychosocial stress and by delivering resources for reducing health risks [46,47]. Therefore, interventions for improving social relations which especially focus on socially deprived groups are likely to help reducing socioeconomic disparities in health.

## Competing interests

The authors declare that they have no competing interests.

## Authors’ contributions

KHJ, RE, JS are principal investigators of the HNR study; OK is the principal investigator of the DFG-funded project ‘Health inequalities and social relationships`. ND participated in conducting the HNR study and coordinated data acquisition for this project. Statistical analyses were done by NV. NV, JK and OK were responsible for interpreting the data and writing the manuscript. All authors contributed to drafting the manuscript and approved the final version. All authors read and approved the final manuscript.

## Pre-publication history

The pre-publication history for this paper can be accessed here:

http://www.biomedcentral.com/1471-2458/12/285/prepub
